# Beyond Diabetes: The Vasculoprotective Effects and Anti-Atherosclerotic Potential of Tirzepatide

**DOI:** 10.3390/ijms262412028

**Published:** 2025-12-14

**Authors:** Łukasz Rzepiński, Anna Tywoniuk, Justyna Jaraczewska, Aysheh Al-Shaer, Michał Wiciński

**Affiliations:** 1 Department of Neurology, 10th Military Research Hospital and Polyclinic, 85-681 Bydgoszcz, Poland; tywoniuk.ania@gmail.com; 2 Department of Clinical Medicine, Faculty of Medicine, University of Science and Technology, 85-796 Bydgoszcz, Poland; 3 Department of Cardiology and Cardiac Surgery, 10th Military Research Hospital and Polyclinic, Powstańców Warszawy 5, 85-681 Bydgoszcz, Poland; jaraczewskaj@gmail.com; 4 Department of Neurology, Institute of Medical Sciences, University of Opole, 45-040 Opole, Poland; aysheh5@gmail.com; 5 Department of Pharmacology and Therapeutics, Faculty of Medicine, Collegium Medicum in Bydgoszcz, Nicolaus Copernicus University, M. Curie Skłodowskiej 9, 85-094 Bydgoszcz, Poland; michal.wicinski@cm.umk.pl

**Keywords:** tirzepatide, type 2 diabetes mellitus, atherogenesis, endothelium, inflammation

## Abstract

Tirzepatide is a long-acting agonist for the glucagon-like peptide-1 and glucose-dependent insulinotropic polypeptide receptors approved for the treatment of type 2 diabetes mellitus, weight management in obese patients, or overweight patients with at least one weight-related comorbid condition. The clinical effects of tirzepatide are demonstrated by improved glycemic control, reduced overall appetite, decreased food intake, and body weight. Several studies indicated that the vasculoprotective effects and anti-atherosclerotic potential of tirzepatide extend far beyond glycemic control. Tirzepatide stimulates the mobilization and function of endothelial progenitor cells, which facilitates vascular repair and mitigates hyperglycemia-induced damage. Tirzepatide enhances the activity of endothelial nitric oxide synthase, reduces the activity of endothelial activation molecules such as intercellular adhesion molecule 1 and vascular cell adhesion molecule 1, promotes vasodilation, and reduces peripheral vascular resistance. Furthermore, the drug inhibits inflammation by suppressing the expression of pro-inflammatory cytokines, such as tumor necrosis factor α, interleukin-1β, and interleukin-6. Moreover, tirzepatide improves lipid profiles by decreasing total cholesterol, low-density lipoprotein cholesterol, and triglycerides, while increasing high-density lipoprotein cholesterol. By improving endothelial function, reducing inflammation, and lowering body weight, tirzepatide lowers both systolic and diastolic blood pressure. This article summarizes the data with special emphasis on the mechanisms underlying the anti-atherosclerotic and vasoprotective effects of tirzepatide, based on studies conducted to date.

## 1. Introduction

Diabetes mellitus type 2 (T2D) is the most common form of diabetes in the world. A few years ago, it mainly affected the elderly, but due to the growing incidence of obesity, the disease has increasingly affected younger people, and now we are facing the epidemic of T2D [1]. In 2021, an estimated 563 million people were affected by T2D, and the age of onset is decreasing [2,3]. Due to the increasing incidence of T2D and its complications, new drugs that can control the course of the disease and reduce its metabolic consequences are highly desirable. Tirzepatide was approved by the U.S. Food and Drug Administration (FDA) in May 2022 and by the European Medicines Agency (EMA) in November 2022 for the treatment of T2D that is not satisfactorily controlled with diet and physical activity. It is the first long-acting polypeptide introduced into therapy that is not only an agonist of the glucagon-like peptide-1 (GLP-1) receptor but also an agonist of the glucose-dependent insulinotropic polypeptide (GIP) receptor. Therefore, the drug improves glycemic control and weight loss compared with the GLP-1 receptor agonist—semaglutide [4,5]. Tirzepatide lowers glycemia in adult patients with T2D, reduces weight, and delays progression to T2D in people with both obesity and prediabetes [6]. Currently, tirzepatide is also recommended as an adjunct to a reduced-calorie diet and increased physical activity for weight management in obese patients and those who are overweight with at least one weight-related comorbid condition (e.g., hypertension, dyslipidemia, obstructive sleep apnea, cardiovascular disease (CVD), prediabetes, or T2D). Furthermore, the efficacy and safety of tirzepatide in children and adolescents, aged 10–17 years, with youth-onset T2D inadequately controlled with metformin and/or basal insulin were confirmed in the SURPASS-PEDS study [4,5,6,7,8,9,10].

Vascular disorders, mainly atherosclerosis, remain the leading causes of death worldwide. Risk factors for atherosclerosis include T2D, obesity, dyslipidemia, hyperglycemia, old age, smoking, and an unhealthy diet. Generally, atherosclerosis leads to arterial narrowing through the formation of plaques containing lipids, fibrin, calcium, and immune cells. Atherosclerosis is a dynamic and measurable process that can be detected in the subclinical stage by imaging and elevated coronary artery calcium scores. Therefore, it is necessary to identify and treat risk factors of atherosclerotic cardiovascular disease (ASCVD) in both secondary prevention and apparently healthy individuals, regardless of age. The recommended algorithms to estimate 10-year fatal and non-fatal CVD risk in apparently healthy people (SCORE2 and SCORE2-OP) do not take into account other potential risk modifiers, such as obesity, obstructive sleep apnea syndrome, and persistently elevated high-sensitivity C-reactive protein (hs-CRP) [11]. Notably, non-diabetic overweight or obese individuals with at least one weight-related complication should undergo effective interventions to reduce the risk of developing T2D and ASCVD.

T2D is a serious risk factor for macrovascular complications (e.g., atherosclerosis, heart attacks, strokes) and microvascular complications (e.g., diabetic nephropathy, diabetic retinopathy, diabetic neuropathy). The pathomechanisms driving diabetes-associated atherosclerosis are complex, involving pathways shared with non-diabetic individuals and characterized by more pronounced immune responses within atherosclerotic plaques. The development of atherosclerosis in T2D is initiated by vascular endothelial dysfunction, followed by lipid accumulation in the subendothelial space and the activation of vascular inflammatory processes [12]. It has been shown that tirzepatide can reduce risk for the development or progression of ASCVD in diabetic and non-diabetic patients with different coexisting metabolic abnormalities through various mechanisms [13]. However, the molecular mechanisms underlying this vascular protection are not fully understood. This article summarizes the data with special emphasis on the mechanisms underlying the anti-atherosclerotic and vasoprotective effects of tirzepatide based on selected studies to date.

## 2. The Pharmacology of Tirzepatide

Tirzepatide is a peptide with the molecular formula C225H348N48O68 and a molecular weight of 4813.45 Da. It contains 39 amino acids in the chain. Due to the presence of the C20 diacid, it is resistant to the action of the dipeptidyl peptidase IV, which is responsible for its long half-life (6 days) and high affinity for albumins. The drug is administered once a week by subcutaneous injection, reaches peak plasma concentrations within 8–72 h, and maintains a stable level after 4 weeks of treatment. The effect of tirzepatide increases proportionally with dose when administered in the abdominal wall, thigh, or upper arm. Absolute bioavailability with such administration is about 80%. The drug binds 99% to plasma albumin. Tirzepatide elimination involves cleavage of the peptide skeleton by proteolytic enzymes, beta-oxidation of the C20 fatty acid, and amide hydrolysis. The half-life of tirzepatide and the elimination half-life of metabolites are about 5 days [8]. Drug metabolites are excreted in urine and feces; no unchanged drug has been detected in urine or stool samples. Tirzepatide increases pancreatic β-cell sensitivity to glucose and enhances insulin secretion in phases 1 and 2, in a glucose-dependent manner. Tirzepatide at a dose of 15 mg increased insulin secretion in phases 1 and 2 by 466% and 302%, respectively. Furthermore, the drug at a dose of 15 mg increased whole-body insulin sensitivity by 63% [9]. Tirzepatide binds to the GIP receptor at a level similar to the natural GIP. In contrast, the affinity of the GLP-1 receptor is about five times weaker than that of the native hormone GLP-1. GIP and GLP-1 receptors are located on the endocrine cells α and β of the pancreas, as well as in the brain, heart, blood vessels, immune system cells (leukocytes), intestines, and kidneys. GIP receptors are also found on adipocytes. Tirzepatide improves glycemic control, reduces fasting and postprandial glucose levels in patients with T2D, increases satiety and fullness, reduces overall appetite, and ameliorates metabolic dysfunction [10,14]. Tirzepatide reduces food intake and decreases body weight by reducing adipose tissue mass, which is believed to improve insulin sensitivity. Age, gender, race, ethnicity, and renal or hepatic impairment do not have a clinically significant effect on the pharmacokinetics of tirzepatide. Tirzepatide exposure increases with decreasing body weight, but the effect of weight loss on the drug pharmacokinetics does not appear to be clinically significant [15]. The main contraindication to using tirzepatide is hypersensitivity to any of its ingredients. However, it is recommended to monitor patients taking oral drugs with a small therapeutic index (e.g., warfarin, digoxin), especially when starting treatment with tirzepatide and after increasing the dose. The dose starts at 2.5 mg once a week via the subcutaneous route and after 4 weeks increases to 5 mg per week subcutaneously. To achieve optimal glycemic control, the dose may be increased by 2.5 mg at intervals of at least four weeks, up to a maximum dose of 15 mg subcutaneously once a week. There is no need to change the dosage in people with liver or kidney failure or due to age, gender, or body weight [16,17,18,19]. Tirzepatide is generally well-tolerated. The most commonly reported side effects are mild to moderate gastrointestinal events, including nausea, diarrhea, and vomiting. Less frequently reported adverse reactions include injection site reactions, hypoglycemia, cholelithiasis, cholecystitis, renal injury, and pancreatitis. Special attention should be paid to the possible exacerbation of diabetic retinopathy symptoms and potentially increased risk of developing thyroid C-cell tumors associated with tirzepatide treatment [20,21].

## 3. Pleiotropic Effects of Tirzepatide on Vascular Endothelium

### 3.1. Endothelium—Structure and Molecular Mechanisms of Dysfunction

The vascular endothelium, a single layer of endothelial cells in the blood vessels, remains an important component in maintaining vascular homeostasis. It regulates blood flow, the diameter of the blood vessels, immune responses of the cells, molecular exchange, proper tissue perfusion, and protection against CVD. Endothelial cells (ECs) exhibit remarkable heterogeneity across different vascular beds, adapting to the specific needs of various organs. They are functioning as a wall, controlling selective transmittance and responding to biochemical and mechanical stimulations to maintain vascular integrity [22,23]. Endothelial function is primarily governed by the balance between vasodilatory and vasoconstrictive factors. Nitric oxide (NO) is produced by endothelial nitric oxide synthase. It is a key mediator of vasodilation, inhibiting platelet aggregation and leukocyte adhesion [24]. Other vasorelaxants are prostacyclin (PGI2) and endothelium-derived hyperpolarizing factor (EDHF). In contrast, vasoconstrictors such as endothelin-1 (ET-1), angiotensin-II (Ang II), several components of the renin–angiotensin system (RAS), and thromboxane A2 (TxA2) regulate vascular resistance and blood pressure [25]. The endothelium also responds to shear stress, the friction exerted by blood flow, which influences gene expression and endothelial phenotype. Laminar shear stress promotes an anti-inflammatory, atheroprotective state, whereas disturbed flow patterns contribute to endothelial dysfunction and atherosclerosis [26]. The endothelium is associated with angiogenesis processes, which are essential for wound healing and tissue regeneration and can be dysregulated in conditions such as diabetic retinopathy [27]. Endothelial dysfunction is characterized by impaired NO bioavailability and increased oxidative stress and inflammation, hallmarks of cardiovascular diseases such as hypertension, atherosclerosis, and the side effects of T2D. Risk factors promoting endothelial damage include aging, hypertension, hyperglycemia, dyslipidemia, and nicotine addiction, leading to vascular complications [28,29,30]. Given its critical role in cardiovascular health, therapeutic strategies targeting endothelial function have gained significant interest in preventing and managing metabolic and vascular diseases.

### 3.2. Tirzepatide Ameliorates Endothelial Dysfunction

Tirzepatide improves vascular endothelial function through several mechanisms. The drug stimulates the mobilization and function of endothelial progenitor cells (EPCs), which facilitates vascular repair and mitigates hyperglycemia-induced damage. Clinical studies have shown that administration of tirzepatide decreases levels of intercellular adhesion molecule-1 (ICAM-1) and hs-CRP, which are well-recognized biomarkers of cardiovascular risk [28]. Tirzepatide has the capacity to increase NO production and enhance the activity of endothelial NO synthase in endothelial cells [31]. By increasing NO bioavailability through improved insulin sensitivity and attenuation of inflammatory signaling, tirzepatide promotes vasodilation and reduces peripheral vascular resistance [27]. It has been shown that chronic inflammation and oxidative stress can cause endothelial dysfunction and atherosclerosis. Tirzepatide reduces inflammatory responses by decreasing the pro-inflammatory cytokines tumor necrosis factor α (TNF-α), interleukin-1β (IL-1β), and interleukin-6 (IL-6) in mouse models [32]. Furthermore, post hoc analyses of data from human clinical trials revealed that tirzepatide may reduce the levels of endothelial activation markers ICAM-1 and vascular cell adhesion molecule 1 (VCAM-1) [33]. Tirzepatide attenuates visceral adipose tissue inflammation by inhibiting the expression of inflammation-related genes, suppressing extracellular signal-regulated kinase signaling, and reducing the infiltration of pro-inflammatory M1 macrophages into the adipose tissue of obese mice [34]. A post hoc analysis of clinical trial data demonstrated that tirzepatide at a dose of 15 mg significantly decreased levels of biomarkers that have been associated with endothelial dysfunction, e.g., hsCRP or ICAM-1 [33,35]. Importantly, tirzepatide improves endothelial function by decreasing systolic and diastolic blood pressure, total cholesterol, low-density lipoprotein (LDL) cholesterol, and triglycerides, while increasing high-density lipoprotein (HDL) cholesterol [36]. GLP-1 receptor agonists, which share a mechanism with tirzepatide, have shown anti-atherosclerotic effects in animal models, including a reduction in vascular inflammation and an improvement in endothelial function primarily through the promotion of angiogenesis and inhibition of oxidative stress [37]. Furthermore, studies indicate that GIP, the other component of tirzepatide’s dual action, possesses endothelial protective properties and can suppress foam cell formation in macrophages by inhibiting the cyclin-dependent kinase 5 pathway [38]. SURMOUNT-1 study revealed tirzepatide’s beneficial impact on atherosclerotic cardiovascular disease risk [25,29]. Beyond diabetes, tirzepatide’s endothelial benefits extend to metabolic syndrome, obesity, and non-alcoholic fatty liver disease (NAFLD). Other areas of interest include neurovascular endothelial protection, with potential applications for tirzepatide in cerebrovascular health and stroke prevention [26,28].

## 4. Tirzepatide and Vascular Inflammation

### 4.1. The Role of Inflammation in Vascular Damage

Processes involved in the deregulation of acute inflammation may lead to uncontrolled chronic inflammation [39]. Chronic inflammation has been shown to contribute to the development of insulin resistance and T2D [40]. Moreover, there is evidence that inflammation can be triggered by diabetes and obesity. Fluctuations in blood sugar levels cause oxidative stress and the release of pro-inflammatory cytokines [41]. As a result, systemic inflammation is associated with endothelial dysfunction in T2D [42]. Inflammatory mechanisms in the blood vessels of individuals with diabetes include the secretion of pro-inflammatory cytokines, vascular hyperactivation of nuclear factor-κB (NF-κB), increased expression of cyclooxygenase, and induced NO synthase, imbalanced expression of pro/anti-inflammatory microRNAs, and dysfunctional stress response systems (sirtuins, Nrf2) [43]. One of the main targets of hyperglycemia is the NF-κB factor. NF-κB inhibits endothelial cell migration induced by high glucose levels [44]. The NF-κB pathway regulates the production of pro-inflammatory cytokines and the recruitment of inflammatory cells that contribute to the inflammatory response [45]. Systemic inflammation leads to the activation and release of the neutrophil extracellular trap and may initiate endothelial damage. Monocytes, in turn, differentiate into macrophages under the influence of T cells. They secrete pro-inflammatory mediators such as TNF-α, IL-6, and interleukin-18 (IL-18), which leads to vascular damage [46]. Importantly, chronic inflammation leads to the onset of insulin resistance and T2D, further supporting the continuation of the inflammation.

### 4.2. Anti-Inflammatory Mechanisms of Tirzepatide

The anti-inflammatory mechanisms of tirzepatide appear to be related to its agonistic effects on GLP-1 and GIP, which are incretin hormones affecting glucose-dependent insulin secretion [47]. Activation of GIP and GLP-1 reduces inflammation and increases anti-inflammatory adiponectin and IGF levels [18]. GLP-1 receptors are highly expressed in many tissues, including pancreatic β cells, lungs, central nervous system, endothelium, gastrointestinal tract, immune cells, and kidneys [48]. Activation of GLP-1 receptors on immune cells inhibits the NLRP3 and NF-κB inflammatory signaling pathways, thereby reducing the production of the inflammatory cytokines IL-6 and TNF-α [49]. In addition, GLP-1 stimulates cAMP and endothelial NO synthase, which have anti-inflammatory effects [50]. Activation of GIP and GLP-1 receptors reduces inflammation caused by excess body fat. GIP is a polypeptide that inhibits gastric emptying by acting on GIP receptors. GIP is linked to the development of insulin resistance (IR) and, at a later stage, to T2D by triggering inflammation in adipocytes. GIPR is highly expressed in the pancreas, central nervous system, adipose tissue, and bones [51]. A study by Varol et al. showed that a GIP analogue can inhibit pro-inflammatory monocytes and neutrophils, thus inhibiting adipose tissue-induced inflammation in a murine model. Furthermore, GIP inhibits the expression of IL-1β, IL-6, and TNF-α [52]. Mantelmacher et al. revealed that agonistic effects on the GIP receptor reduce adipose tissue inflammation through the impact of GIP on myeloid bone marrow cells, which show the presence of GIPR and cause the formation of macrophages in adipose tissue [53]. Although tirzepatide is not an anti-inflammatory drug, several studies have shown that it reduces levels of pro-inflammatory cytokines and endothelial dysfunction by affecting glucose homeostasis [54]. The effect of tirzepatide on inflammation in various tissues and organs is currently of interest to researchers. A study by Guo et al. evaluated the anti-inflammatory effect in the hippocampus of rats and the impact on the development of cognitive impairment. The authors examined mRNA levels of TNF-α, IL-6, and IL-1β, as well as phosphorylated NF-κB expression. This study showed the inhibitory effect of tirzepatide on the increase in these levels and its potential effect on reducing inflammation [55]. Another study on mice examined the impact of oxidative stress and inflammation on the development of nephropathy. Tirzepatide modulates the IL-17 pathway and reduces the expression of pro-inflammatory cytokines (TNF-α, IL-1β, and IL-6) in both serum and renal homogenate [56]. Further confirmation of the anti-inflammatory effect of tirzepatide was provided by a study on the effect of this drug in people with T2D suffering from COVID-19 [54]. In COVID-19, pro-inflammatory cytokines such as ICAM-1, GDF-15, and YKL-40 are activated, resulting in generalized inflammation [57]. Increased release of TNF-α, IL-1β, and IL-6 has also been demonstrated, leading to generalized inflammation. Activation of the GLP-1 receptor inhibits the expression of inflammatory cytokines such as IL-6, IL-1β, TNF-α, and C-reactive protein (CRP) and promotes a milder course of infection [58]. An important element in the development of inflammation is macrophages in adipose tissue, which secrete a significant portion of inflammatory factors. Increased numbers and activity of macrophages in adipose tissue have been demonstrated in people with T2D and in obese individuals [59]. Several studies have addressed the problem of cytokine activation in obese individuals, including TNF-α, whose expression was induced in adipose tissue in obesity and diabetes in rodents [60]. Tirzepatide is a drug with proven weight loss effects. Reducing the amount of adipose tissue reduces its pro-inflammatory effects by decreasing the number of active macrophages and the production of pro-inflammatory cytokines. Patients with type 2 diabetes and obesity were enrolled in the study’s second phase. The effect of tirzepatide was evaluated at doses ranging from 5 to 15 mg. After 26 weeks of tirzepatide treatment at doses of 10 and 15 mg, decreased YKL-40, ICAM-1, leptin, and CRP levels were observed compared with baseline [61]. A study by Packer et al. revealed the effect of tirzepatide on generalized inflammation by examining the level of hsCRP. Tirzepatide or a matching placebo was initiated subcutaneously at a dose of 2.5 mg and increased by 2.5 mg every 4 weeks up to a maximum dose of 15 mg per week after 20 weeks. At 52 weeks, the mean percent decrease in the hsCRP was 38.8% in the tirzepatide group and 5.9% in the placebo group (*p* < 0.001) [62]. Therefore, tirzepatide is considered a drug that limits systemic chronic inflammation. It affects glycemic homeostasis, reduces body weight by reducing the amount of body fat, and decreases the secretion of pro-inflammatory cytokines.

## 5. Tirzepatide Ameliorates Glucose and Lipid Metabolism Disorders

### 5.1. Glucose and Lipid Metabolism in Atherogenesis

Lipids and glucose are the major energy sources in human organisms. Moreover, lipids are the main building blocks of cell membranes. The metabolism of lipids and glucose is often intertwined and has many common points, e.g., the conversion of monosaccharides into fatty acids and cholesterol. Importantly, in a world full of excess food, it is easy to overconsume calories, which, combined with insufficient physical exercise, leads to obesity, fatty liver, and then to metabolic disorders, which are the cause of, among others, diabetes and dyslipidemia [63]. Abdominal obesity combined with IR is characterized by increased production of free fatty acids. Free fatty acids undergo oxidation in mitochondria, where superoxide anions are produced, and the kinase system, NF-kB, is activated, and an inflammatory response develops. In patients with IR, insulin stimulation of the phosphoinositide 3-kinase-dependent glucose transport pathway and NO synthase expression is impaired, and the vasodilatory effect of insulin is reduced [64]. Lipid and glucose metabolism disorders have been shown to be strongly related to the development of atherosclerosis. Population studies have demonstrated that elevated levels of LDL cholesterol and apolipoprotein B (Apo B)100, the main structural protein of LDL, are directly associated with risk for atherosclerosis, cardiovascular events, disability, and even death. Hyperlipidemia and hyperglycemia lead to increased oxidative damage and micro- and macrovascular changes. Atherosclerotic cardiovascular disease is accelerated in people with obesity and diabetes. Dyslipidemia, hyperglycemia, oxidative stress, and inflammation play a role via a variety of mechanisms operative in the artery wall [65].

### 5.2. Tirzepatide in the Struggle with Glucose and Lipid Metabolism Disorders

Recently completed clinical trials have evaluated different active compounds that are designed to reduce body weight, regulate glycemia levels, and stop the process of metabolic abnormalities that are believed to be the cause of cardiovascular disorders. A phase 3, double-blind, randomized, controlled trial demonstrated the safety and efficacy of tirzepatide compared with placebo in reducing body weight and the rate of progression from prediabetes to T2D. This study enrolled 2539 participants with obesity, of whom 1032 also had prediabetes. Participants were assigned in a 1:1:1:1 ratio to receive tirzepatide at once-weekly doses of 5 mg, 10 mg, or 15 mg, or placebo. At 176 weeks, the mean percent change in body weight among the participants who received tirzepatide was −12.3% with the 5 mg dose, −18.7% with the 10 mg dose, and −19.7% with the 15 mg dose, as compared with −1.3% in the placebo group (*p* < 0.001 for all comparisons with placebo). Furthermore, fewer participants in the tirzepatide groups received a diagnosis of T2D than in the placebo group (1.3% vs. 13.3%; *p* < 0.001) [4,5,6].

In the SUPRASS studies evaluating different cohorts of patients with T2D, a beneficial therapeutic effect of the drug on glycemia control, weight loss without a significant hypoglycemic episode, and a reduction in the number of CV complications was demonstrated [4,7,21,66]. SURPASS-3MRI study assessed tirzepatide versus insulin degludec on liver fat content and abdominal adipose tissue in adults with T2D. The study was conducted at 45 medical research centers and hospitals worldwide. The study group consisted of insulin-naive adults with type 2 diabetes, baseline HbA1c of 7.0–10.5% (53–91 mmol/mol), BMI of at least 25 kg/m^2^, and stable weight. Eligible patients were on metformin alone or in combination with an SGLT2 inhibitor for at least 3 months prior to screening. The aim of this substudy was to characterize changes in liver fat content (LFC), volume of visceral adipose tissue (VAT), and abdominal subcutaneous adipose tissue (ASAT) in response to tirzepatide subcutaneous injection once per week (pooled data for 10 mg and 15 mg) or insulin degludec. In this substudy, tirzepatide showed a significant reduction in LFC, VAT, and ASAT volumes compared with insulin degludec. At 52 weeks, the absolute reduction in LFC was significantly greater in the pooled tirzepatide 10 mg and 15 mg groups (−8.09%) than in the insulin degludec group (−3.38%). The reduction in LFC was significantly correlated with baseline LFC, reductions in VAT, reductions in ASAT, and reductions in body weight in the tirzepatide groups. These data provide additional evidence on the beneficial metabolic effects of this novel dual GIP and GLP-1 receptor agonist [67].

The risk for atherosclerosis is increased in patients with diabetes mellitus, but the underlying mechanism is not fully explained. Hyperglycemia and hyperlipidemia are important risk factors in the development of atherosclerosis in patients with diabetes. Endothelial dysfunction has been proposed as an early manifestation of atherosclerosis. Lee et al. assessed the acute consequences of oral glucose loading on endothelium-dependent flow-mediated dilation (EFMD) and endothelium-independent flow-mediated dilation (EIFMD) of the brachial artery using a high-resolution ultrasound technique. The study, carried out on 11 men (aged 59 ± 5 years) with T2D and without chronic complications of diabetes, compared them with 12 healthy volunteers. The study revealed that EFMD was decreased by acute hyperglycemia induced by 75 g oral glucose intake and by acute hypertriglyceridemia induced by high-fat feeding. These results suggest that chronically repeated hyperglycemia and hypertriglyceridemia are implicated in endothelial dysfunction, and may play important roles in the development and progression of vascular complications in diabetes, probably through increased oxidative stress [68]. Among the mechanisms involved in the development of chronic diabetes complications, direct and indirect toxic effects of hyperglycemia on cells can be distinguished. Direct toxicity of glucose to cells is achieved through its effects on gene expression and protein synthesis, its influence on growth, proliferation, and production of extracellular matrix. As a result of glucose autoxidation, reactive oxygen derivatives are formed, leading to oxidative damage. Indirect glucose toxicity includes protein glycation, activation of the polyol pathway, oxidative stress, activation of the hexosamine pathway, and activation of the protein kinase pathway [69]. In T2D and metabolic syndrome, hypertriglyceridemia and a decrease in HDL cholesterol levels, as well as an increase in the small dense LDL fraction, are more frequently observed. These laboratory abnormalities are commonly referred to as atherogenic dyslipidemia and are mainly caused by IR. Small dense LDL have a greater susceptibility to glycation and glycoxigenation, impaired ability to bind to native LDL receptor, ability to damage endothelium, and pass into the subendothelial space. A reduction in HDL cholesterol levels leads to impaired reverse transport of cholesterol from peripheral tissues to the liver and to impaired antioxidant and anti-inflammatory effects of HDL in the endothelium. Tirzepatide has been shown to increase adiponectin, decrease serum alanine aminotransferase, and decrease lipoprotein biomarkers such as apolipoprotein C-III, apolipoprotein B, and large triglyceride-rich lipoprotein particles as well as small low-density lipoprotein particles [18,70,71]. It has been shown that only 13% and 21% of the reduction in IR can be attributed to drug-induced body-weight reduction, suggesting a possible, independent of body-weight reduction, effect of tirzepatide on biochemical pathways related to insulin action [72]. Wang et al. showed that an increased triglyceride-glucose (TyG) index was associated with a higher risk of multi-vessel coronary artery disease (CAD). The study was carried out on 2792 participants with CAD in China. There was a significant relationship between the TyG index and the incidence of multi-vessel CAD observed. The study indicated that TyG, as an estimation index for evaluating IR, could be a valuable predictor of CAD severity, especially in individuals with pre-T2D [73]. In the SURPASS-CVOT study, 13,299 patients with type 2 diabetes aged ≥40 years, with established atherosclerotic cardiovascular (CV) disease, 7% ≤ HbA1c ≤ 10.5%, and body mass index ≥ 25 kg/m^2^ were randomized 1:1 to once weekly subcutaneous injection of either tirzepatide up to 15 mg or dulaglutide 1.5 mg. The primary outcome is time to first occurrence of any major adverse cardiovascular event (MACE), defined as CV death, myocardial infarction, or stroke. SURPASS-CVOT will provide definitive evidence as to the CV safety and efficacy of tirzepatide as compared with dulaglutide, a GLP-1 receptor agonist with established CV benefit [66]. Tirzepatide, as a new molecule capable of controlling blood glucose levels by combining the dual agonism of GIP and GLP-1 receptors, appears to be a future hope in the struggle with glucose and lipid metabolism disorders.

## 6. The Overall Cardiovascular Impact of Tirzepatide

CVDs are the most common causes of death and disability. In 2021, 20.5 million people died from CVDs, accounting for approximately one-third of all deaths worldwide [74]. People with diabetes are at greater risk of developing CVD. Therefore, it is reasonable to use drugs that affect not only glycemia stabilization but also the risk factors for CVD and MACE: cardiovascular death, myocardial infarction, stroke, and unstable angina requiring hospitalization [68,73]. Tirzepatide has been shown to benefit the prevention of cardiovascular and cerebrovascular diseases [75]. Taktaz et al. described the direct mechanisms of the action of tirzepatide on the cardiovascular system. It seems that the cardioprotective effect is associated with the anti-inflammatory effects of the drug and its effects on apoptosis and autophagy [76]. In a study on mice with sepsis-induced cardiomyopathy, Liu et al. demonstrated the protective effect of the drug and improved survival in the group treated with tirzepatide. Tirzepatide inhibits the TLR4/NF-κB/NLR3 pathway, which plays a role in apoptosis and inflammation. In mice treated with tirzepatide, the cardiac protein levels of TNF-α, IL-6, and IL-1β were reduced [32]. Wilson et al. showed the effects of tirzepatide on the levels of inflammatory markers YKL-40 and hsCRP; a marker of endothelial dysfunction, ICAM-1; and leptin, a satiety hormone secreted by adipose tissue [33]. Tirzepatide indirectly improves cardiovascular health by influencing glycemic metabolism, obesity, blood pressure, and lipid profile. Obesity predisposes not only to the occurrence of CVD, but also to IR and T2D. Randomized studies on tirzepatide have shown its effect on weight loss and the reduction in fat tissue. The SURPASS-3 study compared weight loss in people with type 2 diabetes taking tirzepatide and those treated with insulin degludec. In the first group, weight loss was 9.8 kg in subjects receiving 5 mg of tirzepatide and 15.2 kg in those receiving 15 mg of tirzepatide [77]. In phase III of the placebo-controlled study, SURMOUNT-1, tirzepatide was tested in various doses (5, 10, 15 mg) in overweight or obese people without diabetes. At 72 weeks, 15% weight loss was achieved in individuals receiving 5 mg of tirzepatide, 19.5% in those who received tirzepatide at a dose of 10 mg, and 20.9% in those receiving 15 mg of tirzepatide, and 3.1% weight loss was observed in the placebo group (*p* < 0.001 for all comparisons with placebo) [78]. A post hoc analysis of the three-year SURMOUNT-1 trial revealed that tirzepatide treatment was associated with a reduction in the 10-year predicted risk of cardiovascular outcomes and T2D in people with obesity and prediabetes [79]. Tirzepatide has been proven to have a beneficial effect on reducing adipose tissue, not only in diabetics but also in obese people without diabetes. Increased total cholesterol, LDL, triglycerides (TG), and decreased HDL predispose to the development of atherosclerosis. Atherosclerotic plaques form in blood vessels, which can rupture, initiating the coagulation cascade and the possibility of thrombus detachment. They increase the risk of cardiovascular events, including MACE. Tirzepatide has a beneficial effect on the lipid profile, reducing the level of total cholesterol, LDL, and TG and increasing the level of HDL, and this effect is dose-dependent [61]. In the SURPASS-5 study, 475 adults with T2D and inadequate glycemic control with once-daily insulin glargine with or without metformin were randomized in a 1:1:1:1 ratio to receive once-weekly subcutaneous injections of 5 mg, 10 mg, or 15 mg tirzepatide or volume-matched placebo over 40 weeks. Treatment with 15 mg tirzepatide resulted in a decrease in total cholesterol by 12.9%, LDL-C by 15.5%, triglycerides by 24.9% and an increase in HDL by 0.9% [80]. The regulation mechanism of lipids is possibly related to the homeostasis activation of the GIP receptor, which increases blood flow through adipose tissue and promotes adipose tissue lipid uptake [81]. One of the critical risk factors for CVDs is arterial hypertension. Several randomized studies have shown a beneficial effect of tirzepatide on lowering systolic and diastolic blood pressure. The most significant reduction in blood pressure during tirzepatide treatment compared to placebo was achieved in the SURPASS-5 study. In the placebo group, the reduction in mean systolic blood pressure (SBP) was −1.7 mmHg, and diastolic blood pressure (DBP) was −2.1 mmHg. In the tirzepatide group, the reduction in systolic blood pressure ranged from −6.1 to −12.6 mmHg, and diastolic blood pressure from −2.0 to −4.5 mmHg [80]. The effect of tirzepatide on blood pressure is probably related to its impact on improving endothelial function, reducing inflammation, and body weight [80]. GIPR and GLP1R are present in smooth muscle cells and endothelial arterioles [82]. GIP regulates inflammation and leukocyte adhesion via ET-1 while simultaneously dilating blood vessels via NO secretion [83]. GLP-1 increases eNOS pathway activity and nitric oxide production [84]. Interestingly, tirzepatide significantly reduced the risk of major adverse limb events in peripheral artery disease patients with T2D and was associated with lower mortality, stroke, and MACE in this group [85]. An important aspect is also the assessment of tirzepatide in terms of its effect on cardiovascular events, including cardiovascular death, myocardial infarction, stroke, and unstable angina requiring hospitalization. Del Prato et al. aimed to assess efficacy and safety, especially CV safety, of tirzepatide versus insulin glargine in adults with T2D and high cardiovascular risk inadequately controlled on oral glucose-lowering medications. Eligible patients with T2D were treated with any combination of metformin, sulfonylurea, or sodium-glucose co-transporter-2 inhibitor, had a baseline glycated hemoglobin (HbA1c) of 7.5–10.5% (58–91 mmol/mol), body mass index of 25 kg/m^2^ or greater, and established cardiovascular disease or a high risk of CV events. Study participants were randomly assigned (1:1:1:3) to subcutaneous injection of either once-per-week tirzepatide (5 mg, 10 mg, or 15 mg) or glargine (100 U/mL), titrated to reach fasting blood glucose of less than 100 mg/dL. At 52 weeks, mean HbA1c changes with tirzepatide were −2.43% with 10 mg and −2.58% with 15 mg, versus −1.44% with glargine. The percentage of participants with hypoglycemia was lower with tirzepatide (6–9%) versus glargine (19%). Adverse effects such as nausea, diarrhea, decreased appetite, and vomiting were more common in the tirzepatide group than in the glargine group, mainly mild to moderate, and occurred during the dose-escalation phase. The authors estimated the risk of MACE at 0.5. Adjudicated MACE-4 events (cardiovascular death, myocardial infarction, stroke, hospitalization for unstable angina) occurred in 109 individuals and were not increased on tirzepatide compared with glargine [86]. The results of the SURPASS-CVOT study, which is designed to compare tirzepatide with dulaglutide regarding cardioprotection and MACE, are currently awaited [63]. Tirzepatide and its effect on heart disease remain of interest to scientists. Liu et al., in a study conducted on mice with sepsis-induced cardiomyopathy, showed that tirzepatide could reduce susceptibility to ventricular arrhythmia and systolic dysfunction of the heart [32]. In the SUMMIT study, Packer et al. showed that people with obesity and heart failure with preserved ejection fraction (HFpEF) who were treated with tirzepatide had a lower risk of the composite of death from cardiovascular causes or worsening heart failure compared to subjects who received the placebo. In this international trial, 731 obese patients with heart failure, an ejection fraction of at least 50%, receive tirzepatide (up to 15 mg subcutaneously once per week) or placebo for at least 52 weeks. At 52 weeks, the mean increase from baseline in the Kansas City Cardiomyopathy Questionnaire clinical summary score was significantly higher in the tirzepatide group (19.5 ± 1.2) as compared with the placebo group (12.7 ± 1.3) [62]. The cardiac magnetic resonance substudy of the SUMMIT trial demonstrated a reduction in left ventricular mass by 11 g and a decrease in pericardial adipose tissue by 45 mL in the tirzepatide group when corrected for the placebo group [87]. This probably affects the decrease in heart failure exacerbations in the SUMMIT study. Interestingly, it has been shown that patients with T2D and obesity treated with GLP-1 agonists (semaglutide or tirzepatide) had significantly lower risks of dementia, stroke, and all-cause mortality compared with those receiving other antidiabetic drugs, but no difference in the risk of Parkinson’s disease or intracerebral hemorrhage [88]. The ongoing randomized-controlled trial assessing the effect of tirzepatide treatment on coronary atherosclerosis progression in T2D patients, using multidetector computed tomography angiography, will provide the nature of coronary atherosclerosis progression, regression, and stabilization under the influence of dual GLP-1-GIP therapy [89].

Table 1 summarizes the studies evaluating the efficacy of tirzepatide.

### Translational Application Prospects

Tirzepatide has antidiabetic effects with important cardio- and vasculoprotective properties in patients with or without T2D. The drug’s multifaceted action significantly expands its potential for early use in ASCVD. To address these needs, early identification and monitoring of atherosclerosis using recent developments in diagnostic techniques (e.g., intravascular ultrasound, microscopy imaging, magnetic resonance imaging, positron emission tomography, and single-photon emission computed tomography), laboratory testing (e.g., lipoprotein(a), imidazole propionate, and apolipoprotein B), and genetic techniques (e.g., microRNAs, whole-blood gene expression profiles) remain crucial. In the era of personalized medicine, developing a profile of patients with CVD who will most benefit from early tirzepatide initiation, with adjustments to the drug dose and treatment duration, will reduce the global burden of these disorders and comorbidities, as well as polytherapy.

## 7. Conclusions

The aforementioned data showed that tirzepatide has vasculoprotective effects and anti-atherosclerotic potential, extending far beyond glycemic control. By improving vascular endothelial function, inhibiting inflammatory signaling pathways, reducing body weight, lowering blood pressure, and improving lipid profiles, the drug inhibits atherosclerotic processes at various levels. Therefore, tirzepatide has the potential to beneficially impact various components of the metabolic syndrome, significantly reducing the risk of cardiovascular events. Although the studies included in this review differ in methodology, drug dose, study populations, and analyzed endpoints, they indicate clinically important aspects of cardiovascular events prevention in patients with T2D treated with tirzepatide. However, as the frequency of tirzepatide use increases, the adverse effects profile will require special attention, especially with long-term use of high doses of the drug.

## Figures and Tables

**Table 1:** Summary of studies evaluating the efficacy of tirzepatide.

Study	Characteristics of the Subjects	Doses of Tirzepatide	Results
SURPASS 1 [21]	T2D patient inadequately controlled by diet and exercise vs. placebo.	5, 10, 15 mg once a week	↓ HbA1c (%), Weight loss (kg)
SURPASS 2 [90]	T2D patient inadequately controlled by diet and exercise vs. Semaglutide.	5, 10, 15 mg once a week	↓ HbA1c (%), Weight loss (kg)
SURPASS 3 [77]	T2D patient inadequately controlled by diet and exercise vs. once-daily insulin degludec as an add-on to metformin with or without inhibitors of Sodium/Glucose Transporter 2 (SGLT2).	5, 10, 15 mg once a week	↓ HbA1c (%), Weight loss (kg)
SURPASS 4 [86]	T2D patient inadequately controlled by diet and exercise with a high cardiovascular risk vs. insulin glargine.	5, 10, 15 mg once a week	↓ HbA1c (%), Weight loss (kg)
SURPASS 5 [80]	T2D patient inadequately controlled despite treatment with insulin glargine.	5, 10, 15 mg once a week	↓ HbA1c (%), Weight loss (kg)
SURPASS 6 [91]	T2D patient inadequately controlled with basal insulin with or without any combination of up to 2 of the following oral glucose-lowering medications: metformin of at least 1500 mg per day, sulfonylurea, or dipeptidyl peptidase-4 inhibitors.	5, 10, 15 mg once a week	↓ HbA1c (%), Weight loss (kg)
SURPASS-CVOT [66]	T2D patient 40 years or older with established atherosclerotic CV disease, HbA1c (7–10.5%), and BMI of 25 or higher.	up to 15 mg compared to dulaglutide	↓ HbA1c (%), Weight loss (kg), Improves renal function and decreases all-cause mortality
SURPASS-PEDS [7]	Pediatric and adolescent participants with T2D inadequately controlled with metformin or basal insulin, or both.	5, 10 mg once a week or PBO	↓ HbA1c (%), Weight loss (kg)
SURMOUNT-1 [78]	Non diabetic patient with a diagnosis of obesity.	5, 10, 15 mg once a week	↓ body weight (kg) ↓ waist circumference (cm)
SURMOUNT-2 [92]	Adults with obesity and T2D.	10, 15 mg once a week or PBO	↓ body weight (kg)
SURMOUNT-3 [93]	Adults with obesity or overweight who had already lost more than 5%weight following a 12-week intensive lifestyle program.	10, 15 mg once a week or PBO	Tirzepatide improved HRQoL
SURMOUNT-4 [94]	Obesity patients of BMI 30 or higher, or BMI of 27 or higher, and weight-related complications, excluding diabetes.	10, 15 mg once a week for 36 weeks than 10, 15 mg or PBO	↓ body weight (kg)
SURMOUNT-5 [5]	Obesity patients of BMI 30 or higher, or BMI of 27 or higher, and at least one prespecified obesity related complication, and reported at least 1 unsuccessful dietary effort of weight reduction.	TIRZEPATIDE (10 or 15 mg once a week) vs. SEMAGLUTIDE (1.7 or 2.4 mg once a week)	Greater weight loss (kg) in the tirzepatide group.
SURMOUNT-J [95]	Japanese adults with a BMI of 27 or higher, accompanied by two or more obesity related health disorders, or a BMI of 35 or higher, accompanied by one or more obesity related health disorders.	10, 15 mg once a week or PBO	↓ body weight (kg)
SURMOUNT-CN [96]	Chinese adults with a BMI of 24 or higher, accompanied by one or more obesity related health disorders, or a BMI of 28 or higher, excluding diabetes.	10, 15 mg once a week or PBO and lifestyle intervention	↓ body weight (kg)
SYNERGY-NASH [97]	Participants with biopsy-confirmed MASH and stage F2 or F3 (moderate or severe) fibrosis.	5, 10, 15 mg once a week or PBO	Improved MASH resolution without worsening of fibrosis
SUMMIT [62]	Patients with heart failure with preserved ejection fraction (HFpEF) and obesity.	15 mg once a week or PBO	↓ body weight (kg) Improved patient-oriented quality of life
Mimura H et al. [98]	Japanese adult participants with T2D.	5 mg, 10 mg, and 15 mg s.c., 4–52 weeks	Weight loss, ↓ Triglycerides, ↓ HDL-C, ↓ SBP, ↓ DBP, ↓ FSG, ↓ SMBG

Note: ↓ = reduction, HDL-C = high-density lipoprotein, SBP = systolic blood pressure, DBP = diastolic blood pressure, FSG = fasting serum glucose, HbA1c = glycated hemoglobin A1c, SMBG = self-monitored blood glucose, HRQoL = health-related quality of life, PBO = placebo, MASH = metabolic dysfunction-associated steatohepatitis, T2D = diabetes mellitus type 2.

## Data Availability

No new data were created or analyzed in this study. Data sharing is not applicable to this article.

## Abbreviations

CAD	coronary artery disease
CV	cardiovascular
CVD	cardiovascular diseases
GLP-1	Glucagon-like peptide-1
GIP	glucose-dependent insulinotropic polypeptide
ECs	endothelial cells
EPCs	endothelial progenitor cells
ICAM-1	intercellular adhesion molecule 1
VCAM-1	vascular cell adhesion molecule 1
TNF-α	tumor necrosis factor α
IL-1β	interleukin-1β
IL-6	interleukin-6
IL-18	interleukin-18
LDL	low-density lipoprotein
HDL	high-density lipoprotein
T2D	diabetes mellitus type 2
FDA	U.S. Food and Drug Administration
EMA	European Medicines Agency
SBP	systolic blood pressure
DBP	diastolic blood pressure
FSG	fasting serum glucose
HbA1c	glycated hemoglobin A1c
SMBG	self-monitored blood glucose
HRQoL	health-related quality of life
PBO	placebo
MASH	metabolic dysfunction-associated steatohepatitis
IR	insulin resistance
NF-κB	nuclear factor-κB
NO	nitric oxide
PGI2	prostacyclin
EDHF	endothelium-derived hyperpolarizing factor
ET-1	endothelin-1
Ang II	angiotensin-II
RAS	renin-angiotensin system
TxA2	thromboxane A2
NAFLD	non-alcoholic fatty liver disease
Apo B	apolipoprotein B
HFpEF	heart failure with preserved ejection fraction
LFC	liver fat content
VAT	volume of visceral adipose tissue
ASAT	abdominal subcutaneous adipose tissue
MACE	major adverse cardiovascular event
TyG	triglyceride-glucose
EFMD	endothelium-dependent flow-mediated dilation
EIFMD	endothelium-independent flow-mediated dilation
CRP	C-reactive protein
